# Effects of Aminoguanidine on Glomerular Basement Membrane Thickness and Anionic Charge in a Diabetic Rat Model

**DOI:** 10.1155/EDR.2001.225

**Published:** 2001

**Authors:** Dilek Gogas Yavuz, Halil Önder Ersöz, Mürvet Tuncel, Mustafa F. Sargon, Belgin Küçükkaya, Rengin Ahiskali, Sema Akalin

**Affiliations:** 1 Section of Endocrinology and Metabolism Department of Internal Medicine Marmara University Istanbul Turkey; 2 Department of Biochemistry Marmara University Istanbul Turkey; 3 Department of Pathology Marmara University Istanbul Turkey; 4 Department of Anatomy Hacettepe University Ankara Turkey

## Abstract

We investigated the effect of aminoguanidine (AG) administration on GBM thickness, glomerular heparan sulfate (HS) content, and urinary albumin and HS excretion in diabetic rats. After induction of diabetes, female Wistar rats were divided into 2 groups: Group AGDM (n=11) received 1g/L aminoguanidine bicarbonate in drinking water, group DC (n=12) was given only tap water. Control rats received AG (group AGH, n=8) or tap water (group HC, n=8). At the end of a period of 8 weeks, urinary albumin and glycosaminoglycan (GAG) excretion was detected. GBM heparan sulfate distribution and count was determined under the electron microscope. The AGDM group had lower urinary albumin and GAG excretion than diabetic controls. GBM thickness was increased in diabetic rats compared to groups of AGDM and HC. In AGDM group alcian blue stained particle distribution and count in the GBM was similar to healthy controls. In conclusion AG prevents the decrease of anionic charged molecules in the GBM and GBM thickening. This can be one of the mechanisms by which AG decreases albuminuria in diabetic rats.


*Int. Jnl. Experimental Diab. Res.*, Vol. 2, pp. 225–232
Reprints available directly from the publisher
Photocopying permitted by license only

© 2001 OPA (Overseas Publishers Association) N.V.
Published by license under
the Harwood Academic Publishers imprint,
part of Gordon and Breach Publishing,
a member of the Taylor & Francis Group.
Printed in the U.S.A.

### Effects of Aminoguanidine on Glomerular Basement Membrane Thickness and Anionic Charge in a Diabetic Rat Model

DILEK GOGAS YAVUZ[a,*], HALIL ÖNDER ERSÖZ[a], MÜRVET TUNCEL[d], MUSTAFA F SARGON[d], BELGIN KÜÇÜKKAYA[b], RENGIN AHISKALI[c] and SEMA AKALIN[a]

[a]*Section of Endocrinology and Metabolism, Department of Internal Medicine,* [b]*Department of Biochemistry,* [c]*Department of Pathology, Marmara University, Istanbul, Turkey;* [d]*Department of Anatomy, Hacettepe University, Ankara, Turkey*

*(Received 4 March 2001; In final form 12 August 2001)*

**We investigated the effect of aminoguanidine (AG) administration on GBM thickness, glomerular heparan sulfate (HS) content, and urinary albumin and HS excretion in diabetic rats. After induction of diabetes, female Wistar rats were divided into 2 groups: Group AGDM (n = 11) received 1 g/L aminoguanidine bicarbonate in drinking water, group DC (n = 12) was given only tap water. Control rats received AG (group AGH, n = 8) or tap water (group HC, n = 8). At the end of a period of 8 weeks, urinary albumin and glycosaminoglycan (GAG) excretion was detected. GBM heparan sulfate distribution and count was determined under the electron microscope. The AGDM group had lower urinary albumin and GAG excretion than diabetic controls. GBM thickness was increased in diabetic rats compared to groups of AGDM and HC. In AGDM group alcian blue stained particle distribution and count in the GBM was similar to healthy controls. In conclusion AG prevents the decrease of anionic charged molecules in the GBM and GBM thickening. This can be one of the mechanisms by which AG decreases albuminuria in diabetic rats.**

*Keywords*: Aminoguanidine; Albuminuria; Glomerular basement membrane; Anionic charge; Heparan sulfate; Diabetes

*Abbreviations*: AGDM, aminoguanidine treated diabetic rats; DC, diabetic controls; HC, healthy controls; AGH, aminoguanidine treated healthy rats

### INTRODUCTION

Thickening of the glomerular basement membrane (GBM) and albuminuria are the characteristic features of diabetic nephropathy.[1–3] Biochemical alterations of GBM subsequent to increased non-enzymatic glycation of structural proteins and formation of advanced glycosylation end products (AGE) have been invoked in the genesis of an altered size and charge-selective barrier.[4–6] Aminoguanidine, a hydrazine compound that inhibits glycosylation, prevents diabetic complications. It has been shown to decrease the widening of GBM,[7,8] limiting mesangial expansion[8,9] and to reducing albuminuria[9–12] in experimental diabetes. Although we do not yet understand the exact mechanism of action of this compound, our hypothesis is that the benefical effects of AG on diabetic kidney could be by preventing the loss of glomerular anionic charge. The aim of this study is to evaluate the effects of AG on glomerular basement membrane charge selectivity.

For the determination of glomerular anionic change, we have measured the following

*Address for correspondence: Sakizgülü Sok. No: 1-3 D: 15, 81030 Kadikoy, Istanbul, Turkey. Tel.: +90 216 4490347, Fax: +90 216 4280013, e-mail: dyavuz@turk.net parameters: albuminuria, which reflects the defect in glomerular permeability,[13] red blood cell anionic charge (RBCCh) that mirrors the electrostatic charge on the glomerular basement membrane,[14] urine glycosaminoglycan, which is the major elements of glomerular anionic barrier, as an marker of GAG loss from GBM,[15–17] and the glomerular basement membrane heparan sulfate particles distribution and count that represent the charge selective barrier.[1]

### MATERIAL AND METHODS

Forty, 10-week old female Wistar rats were used in the study. Following an overnight fast, rats were made diabetic by the injection of 55 mg/kg streptozotocin (Sigma USA, Mo) in sodium citrate buffer (PH 4.5) *via* the intraperitoneal route. After 1 week of the diabetes induction 11 rats were given Aminoguanidine bicarbonate salt (Sigma cat no: A-7259) ad lib in drinking water at a concentration of 1 gr/L (group AGDM). Remaining rats were given only tap water and were followed as diabetic controls (group DC, $n = 12$). Sixteen rats received an equivalent amount of buffer intraperitoneal and half of them served as healthy control rats (group HC), while the other half were given AG 1 gr/L in drinking water (group AGH).[10, 18]

Water bottles were refilled everyday in all groups of rats. Antidiabetic therapy was not given to any group throughout the study period. Rats were kept in light (12 hours light, 12 hours dark) and temperature (25°C) controlled cages at the Marmara University Animal studies laboratory and fed by standard 20% protein containing rat chow.

Experiments were conducted in accordance with internationally accepted principles for the care and use of laboratory animals and were approved by the institutional committee for animal research.

At the beginning and at the end of the 8 week, rats were placed in metabolic cages for collection of 24-hour urine samples. They were sacrificed with ether anaesthesia and blood samples were collected by cardiac puncture. Kidneys were excised. The right kidney was fixed in phosphate buffered formaldehyde solution for light microscopy and processed in paraffin.

### Electron Microscopic Evaluation

Pieces from the upper pole cortex of the left kidney were fixed in 0,3 mM MgCl and 0,05% Alcian blue containing, phosphate buffered 2.5% glutaraldehyde solution and processed in epon.[19] After being cut and placed on a slide, tissues were stained with methylene blue in order to localize glomeruli by light microscopy. Then, the blocks were trimmed down and 60 nm sections were cut and viewed under the electron microscope.

Basement membrane thickness evaluation was done under ×50,000 magnification on 3 glomeruli for each specimen and at least on 5 plan for each glomerulus by the orthogonal intercept method. Three specimens from each study group were evaluated for electron microscopy.

Alcian blue stained particles were assessed quantitatively in an 1 $\mu m^2$ area at five different plans on three glomeruli for three rats in each group. Also distribution pattern of alcian blue stained particles were evaluated.

### Light Microscopic Evaluation

Kidney cortex samples were fixed in formaldehyde solution and processed in paraffin, 5 μ section were cut and stained according to the critical electrolyte concentration technique with 0,3 M $MgCl_2$ and 0,5% Alcian blue 8 GX (Sigma cat no: A-5268).[20] Alcian blue staining of GBM was evaluated at 5 different sites of at least 20 glomeruli for each specimen. Staining was graded between 0–3+ semi quantitatively (0 = no staining, 1+ = focal, 2+ = diffuse mild, 3+ = prominent).

Also a semi-quantitative assessment of renal damage was performed on periodic acid shiff and methenamine silver sections. The following histological findings were considered: mesangial expansion, interstitial fibrosis, tubular atrophy, arteriolar hyalinosis and interstitial inflammation. Mesangial change was graded from 0–3+ (0 = no change, 1+ = mild changes consisting of an expansion of mesangial matrix or cells, without reduction of capillary lamina; 2+ moderate changes consisting of an expansion of mesangial matrix with a reduction of capillary lamina; 3+ severe changes consisting of an expansion of mesangial matrix or cells with marked occlusions of capillary lamina). Interstitial fibrosis and tubular atrophy were graded from 0–3+ (0 = no change, 1+ changes affected less than 10% of the biopsy specimen, 2+ changes affecting 10–25% of the biopsy specimen, 3+ changes affecting more than 25% of the biopsy specimen). Arterial hylinosis was graded from 0–3+ (0 no hyalin deposit; 1+ hyalin deposit <50% of circumference; 2+ hyalin deposit >50 of circumference; 3+ diffuse). Arteriosclerotic changes leading to reduction of the vascular lumen were graded from 0–4+ (0 no change; 1+ mild thickening, less than the luminal diameter; 2+ wall thickness equal to the diameter of the lumen; 3+ wall thickness greater than diameter of the luminal diameter; 4+ luminal occlusion).[21]

In each specimen, perpendicular axis of at least 8 glomeruli, sectioned at a vertical line transpassing the glomerular tuft, were measured by ocular micrometer and the means of two measurements were averaged.[22] Glomerular capillary areas were calculated with the $\pi r^2$ formula.

Total number of cell nuclei per glomerular cross-section of 8 glomeruli were counted for each specimen.[23]

### Determination of RBCCh

RBCCh was evaluated with a cationic dye Alcian blue 8GX (Sigma cat no: A 5268), according to the method of Levin,[24] with minor modifications as follows: from whole blood samples, coagulated with citrate, platelets and leucocytes were removed by the method of Beutler *et al.*[25] Red blood cells (RBC) were washed three times in saline and resuspended in the buffered solution containing alcian blue at a final concentration of 250 mg/L. After a 30-minute incubation period at 37°C, the RBC suspension was centrifuged and the remaining alcian blue concentration was measured in the supernatant by a Shimatsu 2000 UV spectrophotometer at a wavelength of 650 nm. Intra- and inter-assay coefficients of variation of alcian blue binding were 5.8% and 7.6%, respectively.

### Determination of Urinary GAG

Urinary GAG was measured by a Shimatsu 2000 UV spectrophotometer at a wavelength of 520 nm in 24-hour urine samples, with a colorimetric method described by Jong,[26] using 1.9 dimethyleneblue (Aldrich Chem Co, USA) and bovine kidney heparan sulfate as a standard (Sigma Cat No H7640). Intra- and inter-assay coefficients of variation of urine heparan sulfate were 2.4% and 15%, respectively.

Urine albumin excretion was determined by nephalometric kit by Biobak. Plasma glucose was measured by the glucose oxidase method with a colorimetric kit by Boehringer (Mannheim, Germany) for the Hitachi 705 autoanalyzer. Urine and plasma creatinine was measured by a modified Jaffe method with a colorimetric kit by Biobak (USA, CA).

Creatinine clearance was calculated using the formula U.V/P, where U is the urine creatinine, is the volume and P is the plasma creatinine.

Statistical analyses were performed with an IBM compatible PC by an Instat II program. All of the analyses were made by nonparametric tests. Kruskal-Wallis ANOVA, Mann-Whitney U tests were used for comparisons and Spearman rank test was used for correlation analysis. The results were expressed as mean ± SEM.

### RESULTS

At the end of the 8 week study period, fasting plasma glucose levels were 79,5 ± 11,1 mg/dl for healthy controls and 116,3 ± 19 mg/dl for AG treated healthy rats while diabetic animals had significantly elevated levels (328,8 ± 61 mg/dl for

AGDM and 436,2 ± 66,9 mg/dl for DC rats ($p < 0,05$). RBCCh was found to be lower in diabetic control rats than in the other 3 groups ($p < 0,05$) (Tab. I). Urinary 24-hour albumin excretion (UAE) was elevated in the DC group (277.4 ± 37.5 μg/day, $p < 0,001$) compared with HC group (106 ± 27 μg/day) and AGH (82.4 ± 10.7 μg/day). AG treated diabetic rats (162.5 ± 21.8 μg/day) had lower UAE than diabetic controls ($p < 0,05$), but it was still high compared with healthy controls ($p < 0.05$). Urinary albumin/creatinine ratio showed similar results.

Urinary 24-hour heparan sulfate excretion was 899,4 ± 158,6 μg/day for healthy controls while DC rats had significantly higher levels (2075,6 ± 500 μg/day, $p < 0,05$). AGDM group had lower GAG excretion (1050.6 ± 195 μg/day) than DC rats ($p < 0,05$), and comparable with the both healthy groups. Urinary GAG/creatinine ratio displayed similar results.

Urinary albumin excretion was significantly correlated with plasma glucose ($r = 0.87$, $p < 0.0001$) and urinary heparan sulfate excretion ($r = 0.73$, $p < 0.0001$) in all study group. A negative correlation was observed between RBCCh and plasma glucose ($r = -0.51$, $p < 0.05$), urinary albumin excretion ($r = -0.35$, $p < 0.05$) and urinary heparan sulfate levels ($r = -0.61$, $p < 0.0005$).

In electron microscopic evaluation, GBM thickness was 114,6 ± 4,1 nm for AG treated diabetic rats, 224 ± 20,1 nm for non-treated diabetic rats, 95,7 ± 7,6 nm for healthy controls, and 97,3 ± 4,3 nm for AG treated healthy controls. Diabetic groups had higher GBM thickness than healthy groups ($p < 0,001$) while AG treated diabetic rats had lower values compared with diabetic controls ($p < 0,05$), and non-diabetic animals.

Healthy control rats had a diffuse distribution pattern of alcian blue stained particles in the GBM (Fig. 1a). In untreated diabetic rats, alcian blue stained particles were reduced along the basement membrane and subendothelial depositions of anionic particles were observed compared to the healthy control group (Fig. 1b). AGDM group glomeruli showed a similar distribution pattern of alcian blue stained particles throughout the GBM as healthy controls (Fig. 1c). Also AG treated healthy rats showed a pattern of distribution similar to healthy controls.

Alcian blue stained particle count per 1 μm$^2$ was 90.6 ± 0.6, 36.0 ± 2.4, 93.8 ± 1.1, 98.6 ± 1.7 for AGDM, DC, HC, AGH groups respectively. DC group have significantly lower level than the other groups ($p < 0.005$).

During light microscopic semi-quantitative evaluation of alcian blue staining, diabetic controls (Fig. 2a) displayed less staining than healthy rats (Fig. 2b) ($p < 0.01$). Alcian blue staining of glomeruli in AGDM group (Fig. 2c) was strong than DC group ($p < 0.05$) while it wasn't different than HC group.

Mesangial matrix that was evaluated semi-quantitatively appeared to be expanded in untreated diabetic rats compared with healthy

TABLE I Biochemical parameters of rats at the end of the study period

| | AGDM | DC | HC | AGH |
|---|---|---|---|---|
| Glucose (mg/dl) | 328.8 ± 61* | 436.2 ± 66.9* | 79.5 ± 11.1 | 126.3 ± 19 |
| RBCCh (ng alcian blue/10$^6$ RBC) | 465.5 ± 3.9 | 367.5 ± 32.9† | 423.4 ± 14.8 | 468.6 ± 5.5 |
| Albuminuria (μg/day) | 162.5 ± 21.8‡ | 277.4 ± 37.5§ | 106 ± 27 | 82.4 ± 10.7 |
| Albumin/creatine (μg/mg) | 7.36 ± 1.4‡ | 22.8 ± 7.8§ | 0.42 ± 0.12 | 0.39 ± 0.16 |
| Urinary Heparan sulfate (μg/day) | 1050.6 ± 195 | 2075.6 ± 505§§ | 899.4 ± 158.6 | 956 ± 68.7 |
| Urinary Heparan sulfate/creatine (μg/mg creatine) | 4.31 ± 1 | 28.8 ± 11.4¶ | 2.38 ± 0.9 | 1.16 ± 0.16 |

*$p < 0.05$ *vs.* healthy rats.
†$p < 0.05$ *vs.* other groups.
‡$p < 0.05$ *vs.* healthy controls.
§$p < 0.001$ *vs.* healthy groups, $p < 0.05$ *vs.* AGDM.
§§$p < 0.05$ *vs.* other groups.
¶$p < 0.01$ *vs.* healthy rats, $p < 0.05$ *vs.* AGDM.

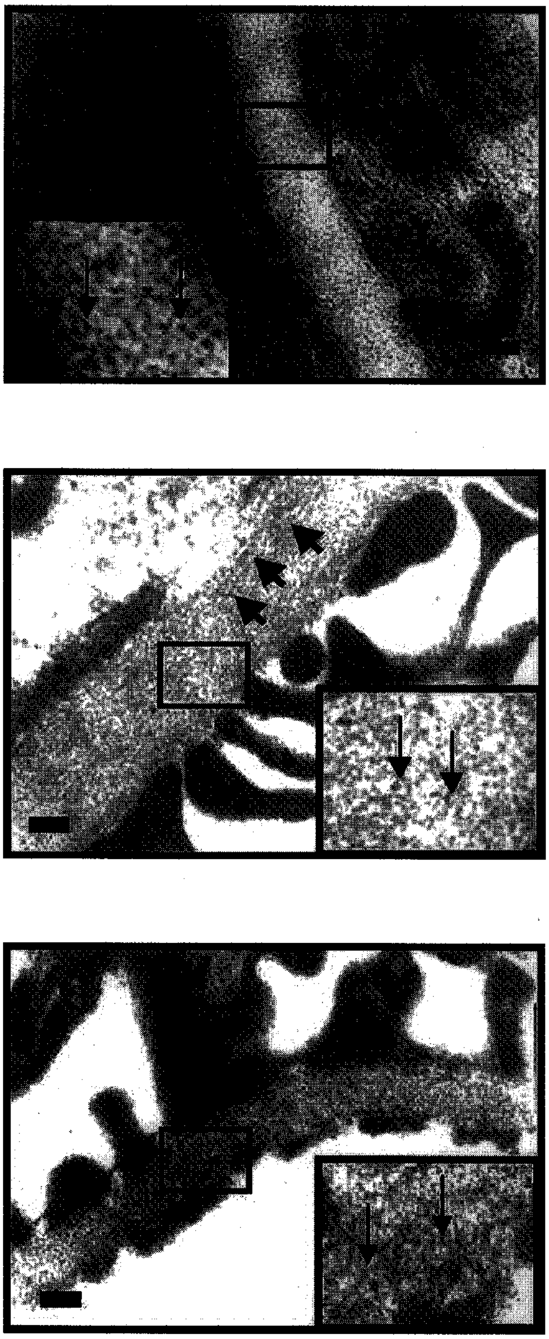

(a) (b) (c)

FIGURE 1 Electron micrographs of glomerular basement membranes from rats: (a) Diffuse distribution pattern of alcian blue stained particles (arrows) in healthy control rat having higher particle number-density (original magnification: ×50 000, bar: 100 nm); (b) Subendothelial depositions (short arrows) of alcian blue stained particles (arrows) in untreated-diabetic rat having lower particle number-density (original magnification: ×50 000, bar: 100 nm); (c) Diffuse distribution pattern of alcian blue stained particles (arrows) of AG treated-diabetic rat having higher particle number-density (original magnification: ×50 000, bar: 100 nm).

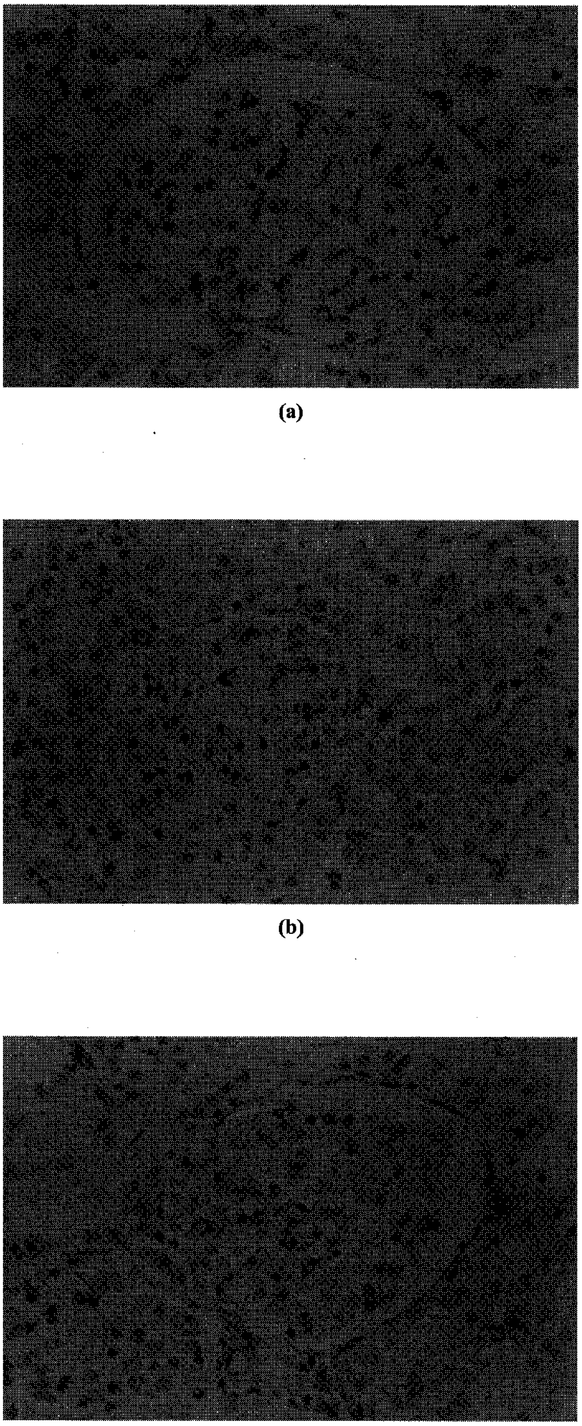

(a) (b) (c)

FIGURE 2 Light microscopic photographs of rat glomeruli stained with alcian blue: Glomerular basement membrane of non-treated diabetic rats displayed less staining of alcian blue (a) than the healthy controls (b). GBM of the AG treated diabetic rats (c) showed a very similar staining pattern with healthy rats. Magnification: x400.

TABLE II Semiquantitative evaluation (number of biopsies in relation to score) for different parameters in renal specimens from diabetic and healthy rats

| | AGDM (n = 11) | | | | DC (n = 12) | | | | HC (n = 8) | | | | AGH (n = 8) | | | |
|---|---|---|---|---|---|---|---|---|---|---|---|---|---|---|---|---|
| | 0 | 1 | 2 | 3 | 0 | 1 | 2 | 3 | 0 | 1 | 2 | 3 | 0 | 1 | 2 | 3 |
| alcian blue staining of GBM | 0 | 2 | 6 | 3 | 2 | 8 | 2 | 0* | 0 | 1 | 2 | 5 | 0 | 0 | 4 | 4 |
| Mesengial matrix expansion | 0 | 2 | 4 | 5† | 0 | 1 | 4 | 6† | 5 | 3 | 0 | 0 | 6 | 2 | 0 | 0 |
| interstitial inflammation | 5 | 6 | 0 | 0 | 2 | 9 | 1 | 0§ | 7 | 1 | 0 | 0 | 8 | 0 | 0 | 0 |
| interstitial fibrosis | 8 | 3 | 0 | 0 | 8 | 3 | 1 | 0 | 7 | 1 | 0 | 0 | 8 | 0 | 0 | 0 |
| tubuler atrophy | 10 | 1 | 0 | 0 | 9 | 2 | 1 | 0 | 8 | 0 | 0 | 0 | 8 | 0 | 0 | 0 |
| arteriolar hyalinosis | 4 | 4 | 3 | 0¶ | 3 | 7 | 2 | 0¶ | 7 | 1 | 0 | 0 | 8 | 0 | 0 | 0 |
| arteriolosclerosis | 3 | 7 | 1 | 0¶¶ | 4 | 6 | 2 | 0¶¶ | 8 | 0 | 0 | 0 | 7 | 1 | 0 | 0 |

*p < 0,0001 *vs.* other groups.
†p < 0,0001 diabetic rats *vs.* healthy rats.
§p < 0,05 *vs.* healthy groups.
¶p < 0,001 diabetic rats *vs.* healthy rats.
¶¶p < 0,05 diabetic rats *vs.* healthy rats.

controls ($p < 0,001$). AG treated diabetic rats had expanded matrix similar with diabetic controls. Basement membrane thickness, evaluated semiquantitatively by light microscopy, was also found to be greater in the diabetic controls compared with healthy control and AG treated diabetics ($p < 0.01$) (see Tab. II).

Total glomerular cell counts were $26,9 \pm 1.6$ for AGDM; $31,0 \pm 1,1$ for DC; $26,6 \pm 1,0$ for HC and $22,6 \pm 1,0$ for AGH groups. The cell count of diabetic controls was significantly higher than that of healthy rats ($p < 0,001$). AG treated diabetic rats had a lower cell count than diabetic controls ($p < 0,01$). Glomerular diameters were $42.2 \pm 0.3\,\mu m$, $46.3 \pm 0.8\,\mu m$, $40.4 \pm 0.6\,\mu m$, and $40.3 \pm 1.0\,\mu m$ for AGDM, DC, HC and AGH groups respectively. Diabetic rats had a higher diameter and surface area compared with healthy animals ($p < 0.05$). AG treated diabetic rats and untreated diabetic rats had a similar glomerular diameter.

### DISCUSSION

The results of this study demonstrate that AG reduces albuminuria and prevents glomerular basement membrane thickening in STZ induced diabetic rats. Over the 8-week study, there was a significant increase in urinary albumin excretion in diabetic rats. In contrast AG therapy retarded the Increase in albuminuria in diabetic rats, resulting in levels similar to those observed in control rats at 8-wk. These results are in concordance with the findings of Ellis *et al.*, who have shown a decrease in urinary albumin excretion and widening of the GBM in STZ induced Lewis rats by AG treatment,[7] and Edelstein *et al.*, who have reported that AG ameliorates albuminuria in diabetic hypertensive rats.[11] On the other hand, there are also conflicting results in the literature. In a type 2 model of diabetes, Yamauchi *et al.*, have shown that AG treatment has no effect on UAE although it reduces GBM thickness.[27] Soulis *et al.*, have reported that the increased glomerular basement membrane thickness in diabetic rats is not affected by aminoguanidine, irrespective of duration or timing of therapy.[8]

Our study confirms that urinary GAG excretion is elevated in non-treated diabetic rats as shown before in diabetic rats by Reddi,[15,16] and in diabetic humans by McAuliffe.[17] We observed a significant decrease in urinary GAG excretion in AG treated diabetic rats compared with non-treated diabetic rats. Urinary GAG excretion was significantly correlated with albumin excretion in all study groups. This might be indicate that increased loss of proteoglycans from diabetic kidneys is prevented by aminoguanidine treatment. This is the first report of such an effect.

This study also shows a significant quantitative reduction of alcian blue binding to RBC in untreated diabetic rats. Aminoguanidine treated diabetic rats have similar levels of alcian blue binding to RBC as healthy rats. As alcian blue binding of RBCs is an expression of the anionic charge of the RBC surface,[14,24] this result indicates that RBC charge is reduced in non-treated diabetic rats while it is protected in AG treated diabetics. RBCCh measurements were negatively correlated with urinary albumin and GAG excretion in all study groups. Those results are in agreement with Gambaro's findings who has suggested that RBCCh could mirror the negative charge of the glomerular basement membrane.[14,24] AG treatment seems to protect the anionic content of both erythrocyte and glomerular basement membrane.

Our results suggest that treatment of diabetic rats with AG prevents this loss of glycosaminoglycans from the GBM. As it has been proposed before that an increased loss of glycosaminoglycans from the GBM alters glomerular charge selectivity, leading to loss of albumin in the urine.[15–17,28]

Results of biochemical parameters are in agreement with electron microscopic and light microscopic findings. In electron microscopic examination total number of alcian blue stained particles in $1\,\mu m^2$ area on the GBM was decreased in untreated diabetic rats compared with healthy animals. On the other hand, AG treated diabetic rats had a similar counts of anionic particles with healthy rats.

Since $Mg^{++}$ ions, in concentrations used in this study, displace carboxylic groups but not sulphate groups, the alcian blue stained structures represent anionic sites of sulphated glycosaminoglycans, primarily heparan sulfate in the glomeruli.[29] As alcian blue only stains the sulphated GAGs, loss of GAGs from the GBM could conceivably be due to either decreased production of sulphation, or adherence to the structural proteins. Another explanation for reduction of alcian blue stained particles could be thickening of the GBM itself. As GBM widens, the density of the particles in $1\,mm^2$ area seems to be decreased.

Besides the preservation of anionic charge, there are many suggested mechanisms explaining favorable effects of AG. Sugimoto *et al.*, have shown that carboxymethyllysine possibly enhances the expression of inducible nitric oxide synthase by stimulating the expression of TNF-$\alpha$ in diabetic rat glomeruli. Treatment with AG reduces the expression of TNF-$\alpha$ and intraglomerular $NO_2/NO_3$ production (30). The study of Osicka *et al.*, indicates that AG prevents diabetes induced changes in lysosomal processing (31). Most remarkable finding is that AG treatment prevented the rise in glomerular protein kinase C activity. Prevention of albuminuria by aminoguanidine has also been associated with the normalization of glomerular protein kinase C (32). In the view of these findings the exact mechanism still remains to be elucidated.

In conclusion, AG treatment reduces urinary albumin excretion, prevents GBM thickness and protects from the loss of GBM anionic content in diabetic rats. Preservation of anionic charges of the GBM seems to be one of the mechanisms by which aminoguanidine ameliorates the albuminuria in diabetic rats.

### Acknowledgements

This study was supported by a Marmara University Research Fund Grant (2-1997) and the Eczacibaşi Scientific Research Award Fund.

### References

[1] Chakrabarti, S., Ma, N. and Sima, F. F. A. (1989). Reduced number of anionic sites is associated with glomerular basement membrane thickening in the diabetic BB-rat. *Diabetologia*, **32**, 326–28.

[2] Osterby, R. (1992). Glomerular structural changes in type 1 (insulin dependent) diabetes mellitus: causes, consequences and prevention. *Diabetologia*, **35**, 803–812.

[3] Mauer, M. S., Steffes, M. W., Ellis, E. N., Sutherland, D. E. R., Brown, D. M. and Goetz, F. C. (1984). Structural-functional relationships in diabetic nephropathy. *J. Clin. Invest.*, **74**, 1143–55.

[4] Brownlee, M. (1992). Glycation products and the pathogenesis of diabetic complications. *Diab. Care*, **15**, 1835–43.

[5] Klein, D. J., Oegama, T. R. and Brown, D. M. (1989). Release of glomerular heparan-35 $SO_4$ proteoglycan by heparan from glomeruli of streptozotocin-induced diabetic rats. *Diabetes*, **38**, 130–39.

[6] Choronis, A. S., Reger, L. A., Dege, J. E., Kauz-Koliakas, K., Furcht, L. T., Wohlhueter, R. M. and Tsilibarg, F. C. (1990). Laminin alterations after *in vitro* nonenzymatic glycosylation. *Diabetes*, **39**, 807–814.

[7] Ellis, E. N. and Good, B. H. (1991). Prevention of glomerular basement membrane thickening by aminoguanidine in experimental diabetes mellitus. *Metabolism*, **40**, 1016–1019.

[8] Soulis, T., Cooper, M. E., Vranen, D., Bucala, R. and Jerums, G. (1996). Effects of aminoguanidine in preventing experimental diabetic nephropathy are related to the duration of treatment. *Kidney Int.*, **30**, 627–634.

[9] Sugiyama, S., Myata, T., Hanse, K., Lida, Y., Tsioyuki, M., Tanaka, H. and Maeda, K. (1996). Advanced glycation end products in diabetic nephropathy. *Nephrol. Dial. Transplant*, **11** (suppl. 5), S91–S94.

[10] Soulis Liporata, T., Cooper, M. E., Papazoglu, D., Clarke, B. and Jerums, G. (1991). Retardation by aminoguanidine of development of albuminuria, mesangial expansion and tissue fluorescence in the streptozotocin-induced diabetic rat. *Diabetes*, **40**, 1328–54.

[11] Edelstein, D. and Brownlee, M. (1992). Aminoguanidine ameliorates albuminuria in diabetic hypertensive rats. *Diabetologia*, **35**, 96–97.

[12] Hanssen, K. F. (1998). Making sense of advanced glycation end products (AGE). *Int. Diab. Monitor*, **10**, 1–5.

[13] Hanssen, P. M., Mathiensen, E. R., Kofoed-Enovoldsen, A. and Deckert, T. (1995). Possible effect of angiotensin converting enzyme inhibition on glomerular charge selectivity. *J. Diab. Compl.*, **9**, 158–162.

[14] Gambaro, G., Baggio, B., Cicerello, E., Mastrosimone, S., Marzaro, G., Basatti, A. and Crepaldi, G. (1988). Abnormal erythrocyte charge in diabetes mellitus, link with microalbuminuria. *Diabetes*, **37**, 745–48.

[15] Reddi, A. S. (1991). Prevention of albuminuria by captopril in diabetic rats. *Gen. Pharmacol.*, **22**, 323–328.

[16] Reddi, A. S., Ramamurti, R., Miller, M., Dhuper, S. and Lesker, N. (1991). Enalapril improves albuminuria by preventing glomerular loss of heparan sulfate in diabetic rats. *Biochem. Med. Metab. Biol.*, **45**, 119–131.

[17] Mc Auliffe, A. V., Fischer, E. J., Mclennon, S. V., Yue, D. K. and Turtle, J. R. (1996). Urinary glycosminoglycan excretion in NIDDM subjects. Its relationship to albuminuria. *Diab. Med.*, **13**, 758–63.

[18] Soulis, T., Cooper, M. E., Sastra, S., Thallas, V., Panagiotopoulos, S. and Bjerrum, O. J. (1997). Relative contributions of advanced glycation and nitric oxide synthase inhibition to aminoguanidine-mediated renoprotection in diabetic rats. *Diabetologia*, **40**, 1141–51.

[19] Raele, E., Luciano, L. and Kühn, K. (1985). Cationic dyes reveal proteoglycans on the surface of epithelial and endothelial kidney cells. *Histochemistry*, **82**, 513–518.

[20] Schumacher, U. and Adam, E. (1994). Standardization of staining in glycosaminoglycan histochemistry: alcian blue it's analogues and diamine methods. *Biothecnic and Biochemistry*, **69**, 18–24.

[21] Bertoni, T., Gambara, V. and Remuzzi, G. (1996). Structural basis of diabetic nephropathy in microalbuminuric NIDDM patients: a light microscopic study. *Diabetologia*, **39**, 1625–28.

[22] Pirani, C. L. (1989). Evaluation of kidney biopsy specimens in renal pathology, Edited by Tisher, C. C. and Brenner, B. M., Philadelphia, Lippincott company, pp. 1–31.

[23] Mauer, S. M. W., Ellis, E. N., Sutherland, E. R., Brown, D. M. and Goetz, F. C. (1984). Structural-functional relationship in diabetic nephropathy. *J. Clin. Invest.*, **74**, 1143–1455.

[24] Levin, M., Smith, C., Walters, M. D. S., Gascoine, P. and Barratt, T. M. (1985). Steroid responsive nephrotic syndrome: a generalised disorder of membrane negative charge. *The Lancet*, **2**, 239–242.

[25] Beutler, E., Weat, C. and Blume, K. G. (1976). Removal of leucocytes and platelets from whole blood. *J. Lab. Clin. Med.*, **88**, 328–333.

[26] Jong, J. G. N., Wavera, R. A., Laarakkers, C. and Poorthula, B. J. H. M. (1989). Dimethylene blue based spectrophotometry of glycosaminoglycans in untreated urine: a rapid screening procedure for mucopolysaccharidosis: *Clin. Chem.*, **35**, 1472–1477.

[27] Yamauchi, A., Taka, I., Makata, Z., Hakamato, S., Ohani N. and Kiguchi, H. (1997). Effects of aminoguanidine on serum AGE, urinary albumin excretion, mesangial expansion and glomerular basement membrane thickening in otsuka long evans tokashima fatty rats. *Diabetes. Res. Clin. Pract.*, **34**, 127–130.

[28] Reddi, A. S. (1990). Glomerular and urinary glycosaminoglycans in diabetic rats. *Clinica Chemica Acta*, **189**, 211–220.

[29] Cook, H. C. (1990). Carbohydrates: Theory and Practice of Histologic techniques, edited by London, *Curchill Livinstone Inc.*, pp. 177–237.

[30] Sugimoto, H., Shikata, K., Wada, J., Horiuchi, S. and Makino, H. (1999). Advanced glycation end products-cytokine-nitric oxide sequence pathway in the development of diabetic nephropathy: aminoguanidine ameliorates the overexpression of tumour necrosis factor-a and inducible nitric oxide syntase in diabetic rat glomeruli. *Diabetologia*, **42**, 878–886.

[31] Osicka, T. M., Kiriazis, Z., Pratt, L. M., Jerums, G. and Comper, W. D. (2000). Ramipril and aminoguanidine restore renal lysosomal processing in streptozotocin diabetic rats. *Diabetologia*, **44**, 230–236.

[32] Osicka, T. M., Yu, Y., Panagiotopoulos, S., Clavant, S. P., Kiriazis, Z., Pike, R. N., Pratt, L. M., Russo, L. M., Kemp, B. E., Comper, W. D. and Jerums, G. (2000). Prevention of albuminuria by aminoguanidine or ramipril in streptozotocin-induced diabetic rats is associated with the normalization of glomerular protein kinase C. *Diabetes*, **49**, 87–93.

